# Morphometric Blastocyst Assessment: A Retrospective Study Examining the Relationship Between Blastocyst Diameter and Area and Pregnancy Outcomes in Assisted Reproduction Technology Cycles

**DOI:** 10.3390/jcm14082827

**Published:** 2025-04-19

**Authors:** Romualdo Sciorio, Pier Francesco Greco, Luca Tramontano, Giuseppe Gullo, Ermanno Greco

**Affiliations:** 1 Fertility Medicine and Gynaecological Endocrinology Unit, Department Woman Mother Child, Lausanne University Hospital, 1011 Lausanne, Switzerland; 2 Reproductive Medicine & IVF Unit, Royale Hayat Hospital, Hawalli 32002, Kuwait; 3 Villa Mafalda, Centre for Reproductive Medicine, 00199 Rome, Italy; 4 Département de Gynécologie-Obstétrique, Réseau Hospitalier Neuchâtelois, 2000 Neuchâtel, Switzerland; luca.tramontano30@gmail.com; 5 Azienda Ospedaliera Ospedali Riuniti (AOOR) Villa Sofia Cervello, IVF Public Center, University of Palermo, 90133 Palermo, Italy; gullogiuseppe@libero.it; 6 Department of Obstetrics and Gynecology, UniCamillus, International Medical University, 00199 Rome, Italy

**Keywords:** embryo culture, morphometric blastocyst assessment, blastocyst area, blastocyst diameter, single blastocyst transfer, artificial intelligence, deep learning, implantation rate, clinical pregnancy outcome

## Abstract

**Objective**: In assisted reproductive technology (ART), achieving a successful pregnancy requires optimizing an embryo culture and selecting the single embryo with the highest implantation potential, capable of resulting in a healthy pregnancy. The primary goal of this study was to determine the correlation between the blastocyst area and diameter and pregnancy outcomes in ART treatments. **Methods**: In this study, the blastocyst diameter and area were measured to determine whether these morphometric features could predict pregnancy outcomes in couples undergoing ART with ICSI. This is a retrospective trial analyzing 665 patients who underwent an ART cycle with the transfer of a single blastocyst on day 5. **Results**: Both morphometric features assessed were significantly associated with implantation and ongoing pregnancy outcomes. Our results showed that the implantation rate (IR) and ongoing clinical pregnancy rate (CPR) were significantly higher with a blastocyst area ≥ 25,000 µm^2^ compared to <25,000 µm^2^ (IR: 69.8% versus 47.9%, *p* < 0.001; CPR: 65.5% versus 45.9% *p* < 0.001). Additionally, a blastocyst diameter ≥ 170 µm resulted in a significantly higher IR and CPR compared to embryos with a diameter < 170 µm (IR: 68.8% versus 36.6%, *p* < 0.001; CPR: 66.3 versus 35.7%, *p* < 0.001). **Conclusions**: Blastocyst morphometric variables, being objective and measurable, are not subject to intra-operator variability and may serve as promising predictors of embryo viability and ongoing pregnancy success. These morphometric assessments could assist embryologists in selecting the embryo with the highest implantation potential from a cohort, as well as identifying those with a reduced chance of generating a successful pregnancy.

## 1. Background

Since the birth of the first baby conceived through IVF in 1978, thanks to the groundbreaking efforts of Patrick Steptoe and Robert Edwards, ART has made significant progress, enabling millions of infertile individuals to have children [1,2,3]. Embryologists in the field continue to refine selection methods to identify the single embryo with the highest likelihood of implantation and live birth while minimizing the occurrence of multiple pregnancies, which pose risks to both mother and child [4,5,6]. The transfer of embryos on day 5, following a prolonged culture to the blastocyst stage, allows for the selection of embryos that have activated their genome, as opposed to those at the cleavage stage, potentially offering a greater chance for successful implantation. However, there is a still ongoing debate regarding the methods used for embryo selection. While morphological evaluation remains the primary approach, it is subject to significant variability between different operators [7,8,9,10]. The novel application of a continuous embryo assessment in combination with a time-lapse monitoring (TLM) technology has enabled embryologists to explore new features of embryo development, such as morphometrics and morphokinetics, from fertilization to the blastocyst stage. However, this approach remains expensive and is not accessible to many IVF centres worldwide. Additionally, a recent study published in *The Lancet* showed no benefit in pregnancy outcomes with the use of a time-lapse. In ta study by Kieslinger and co-workers [11], around 1700 couples were randomly assigned to a double-blind trial conducted across 15 ART units in The Netherlands. The research involved three groups: the time-lapse routine group (TLR), the time-lapse group with embryo viability assessment using the EEVA time-lapse selection algorithm (TLE), and a control group with a standard embryo selection and interrupted culture. Surprisingly, the results revealed no significant differences between the three groups regarding pregnancy outcomes following a fresh single embryo transfer at the cleavage stage on day 3 of embryo development (36.8% in the TLR group, 38.2% in the TLE group, and 37.8% in the control group). These results might be due to the fact that embryo transfers were performed on day 3 and rather than day 5, or other confounding variables that were not deeply explored in the study. Furthermore, the one-year cumulative ongoing pregnancy rate did not differ significantly between the groups: 50.9% in the TLR group, 50.8% in the TLE group, and 49.4% in the control group. A similar randomized, multicenter, controlled study, published by Bhide and colleagues, also found comparable results. The authors concluded that in patients undergoing ICSI or IVF cycles, the use of TLM systems for embryo culture and selection does not significantly improve the percentage of live births compared with a standard culture without TLM [12]. Despite these findings, morphological assessment of preimplantation embryo development remains the primary method in ART, as it is considered non-invasive and does not require expensive equipment. However, there is still an active debate regarding which morphological parameters, including blastocyst expansion, hatching, the trophectoderm (TE) quality, or inner cell mass (ICM) quality, are most indicative of a successful pregnancy [13]. Additionally, degenerated areas within the ICM have been correlated with poor pregnancy outcomes. Some authors have suggested that blastocyst expansion is an important factor in embryo selection [14,15,16,17]. However, research by Hill and colleagues revealed that only the woman’s age and the quality of the TE were strongly linked to implantation and live birth [18]. As a result, it seems evident that the existing blastocyst grading systems should be improved by integrating more objective criteria. Published reports have attempted to establish more consistent and analytical methods for embryo selection, including the quantitative measurement of blastocysts, knows as morphometry assessment. In particular, the measurements of the blastocyst diameter, the ICM area, and the number of TE cells have been studied as morphometric features correlating with blastocyst quality and viability [17,19,20,21,22,23,24,25]. Our group [19] previously reported that women who achieved a positive pregnancy following ART had significantly larger blastocyst diameters (184 μm) compared to non-pregnant women (160 μm). Similarly, when measuring the blastocyst area, we found that pregnant women had an average area of 26,099 μm^2^, compared to 22,251 μm^2^ in non-pregnant women. These results helped establish the cutoff values used in the current study. However, despite the promising and encouraging reports on morphometric variables, the results remain inconclusive and cannot replace a morphology assessment. Therefore, to evaluate the predictive value of the above-mentioned morphometric features for ART success, we conducted a retrospective study to investigate whether the measurement of blastocyst area and diameter could predict the implantation rate (IR) and clinical pregnancy rate (CPR) following the transfer of a single blastocyst.

## 2. Materials and Methods

This retrospective investigation involved 665 ICSI treatments performed at the IVF Department of Royale Hayat Hospital in Kuwait between October 2018 and July 2023. Participants were fully informed about the study and provided their written consent to participate. All treatments included in this study were first ICSI cycles for couples undergoing ART with their own gametes, each involving an elective fresh single blastocyst transfer (eSBT) performed on the morning of day 5. Morphometric blastocyst assessment was performed approximately 110 to 116 h after ICSI. The ongoing clinical pregnancy rate was confirmed by an ultrasound examination of the gestational sac, with the presence of a fetal heartbeat at seven weeks of gestation. The implantation rate was determined by calculating the ratio of embryos that were successfully implanted to the total number of embryos transferred. For this study, all blastocyst images were captured using Chronos Software version 1.6 with a CCD camera (CS230B, Olympus, Tokyo, Japan) to measure morphometric features, including blastocyst diameter and area. All measurements were performed by the consultant embryologist (RS). The data obtained were associated with IR and CPR.

### 2.1. Controlled Ovarian Stimulation

All participants in this study received ICSI treatment. Women underwent controlled ovarian stimulation using a GnRH agonist (subcutaneous buserelin 0.5 ml daily) or a GnRH antagonist (subcutaneous Cetrorelix 0.5 mg daily, Merck Serono, Darmstadt, Germany). The long protocol for down-regulation involved subcutaneous buserelin, while the GnRH antagonist Cetrotide was administered daily, starting on day six of menstruation. Ovarian stimulation was carried out using either Gonal F (Merck Serono, Darmstadt, Germany) or Menopur (Ferring, Saint-Prex, Switzerland), depending on the individual characteristics of each patient. Follicular growth was monitored through transvaginal ultrasound, and ovulation was triggered when three follicles reached 18 mm or larger. Each patient received hCG (Ovitrelle 0.25 mg, Merck Serono, Darmstadt, Germany). Oocyte retrieval was conducted under conscious sedation with transvaginal ultrasound guidance, 35 h after the Ovitrelle injection.

### 2.2. Oocyte Collection, Fertilization and Embryo Assessment

The procedure for oocyte collection and embryo culture has been previously described by Sciorio and colleagues [23]. In brief, cumulus-oocyte complexes (COCs) were separated from follicular fluid, rinsed in pre-equilibrated G-IVF medium (Vitrolife, Gothenburg, Sweden), and transferred to 0.5 mL pre-equilibrated G-IVF (Vitrolife, Sweden). The COCs were incubated at 37 °C in an atmosphere containing 6.0% CO_2_, 5.0% O_2_, and nitrogen balance in a Hera Cell 240 incubator (Thermo Fisher Scientific, Waltham, MA, USA). All media were overlaid with mineral oil (Vitrolife, Gothenburg, Sweden) to prevent evaporation and were equilibrated overnight in the same conditions. Sperm for the ICSI procedure were collected by masturbation and processed using a standard method described by Bourne and collaborators [26]. All COCs were cultured in G-IVF medium until the ICSI procedure, which was performed approximately 38–40 h after the Ovitrelle injection. Oocytes were examined for the presence of two pronuclei 16–19 h after ICSI. Normally fertilized pronuclear-stage embryos were transferred to the G210 InviCell incubator (CooperSurgical, Ballerup, Denmark) for further culture. The in vitro environment was maintained at 6.0% CO_2_, 5.0% O_2_, and nitrogen balance at 37 °C. Each pronuclear stage embryo was cultured using G-series sequential medium (Vitrolife, Gothenburg, Sweden) as follows: fertilized oocytes were transferred in a 10 µL drop of G-1™ media (Vitrolife, Gothenburg, Sweden), covered by mineral oil (Vitrolife, Gothenburg, Sweden). Embryos were scored on days 2, 3, and 5 under a high-power inverted microscope (Olympus, Olympus, Tokyo, Japan). On day 3, embryos were morphologically assessed for cell number, regularity of cleavage, and the degree of fragmentation, according to the scoring system described by Cutting and co-workers [27]. Embryos were then transferred to a 10 µL droplet of G-2^TM^ (Vitrolife, Gothenburg, Sweden) for extended culture to blastocyst stage. Extended culture to the blastocyst was carried out if at least four good quality embryos were present on day 3 (6 to 10 symmetrical blastomeres per embryo, less than 10% fragmentation). All patients included in this study underwent embryo transfer on day 5, at the blastocyst stage. Elective SBT was performed using a transfer medium containing hyaluronan and recombinant human albumin (EmbryoGlue™, Vitrolife, Sweden). Any remaining good quality blastocysts were cryopreserved using a Vitrification protocol as described by others [28,29,30] or were discarded if not deemed suitable for freezing.

### 2.3. Blastocyst Morphometric Evaluation

**Figure 1:**
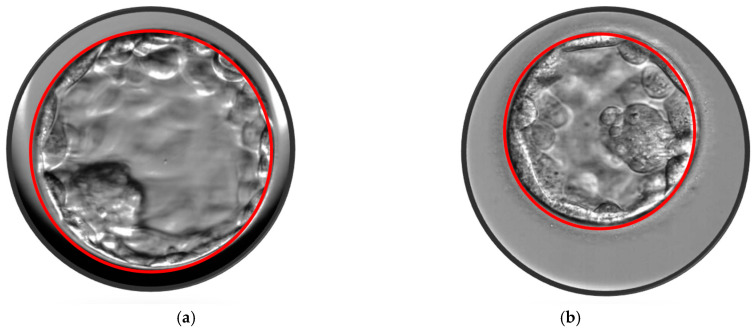
(**a**) Blastocyst area 33,549 μm^2^. (**b**) Blastocyst area 19,704 μm^2^.

**Figure 2:**
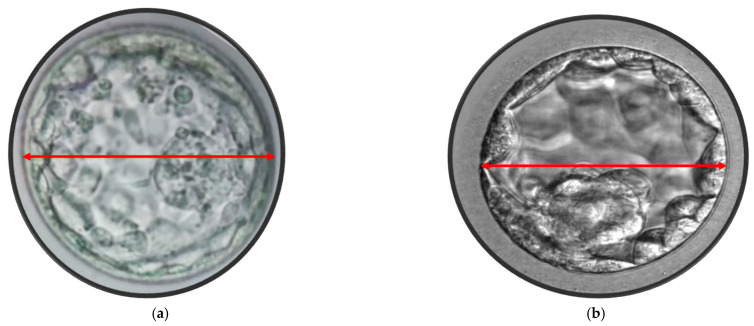
(**a**) Blastocyst maximum diameter 178 μm. (**b**) Blastocyst maximum width 148 μm.

After five days of development, human embryos reach the blastocyst stage, which consists of two differentiated cell types: the ICM and TE cells. In this study, blastocyst evaluation was based on the degree of the blastocoel cavity expansion, as well as the quality and cohesion of ICM and TE, following the Gardner’s scoring system [31]. Only blastocysts with a score ≥ 2, excluding those graded as CC, were selected for eSBT or cryopreservation. On the morning of day 5, before the embryo transfer, approximately 110–116 h after ICSI, morphometric assessments of all blastocysts were performed at 400× magnification using an inverted microscope (IX71, Olympus, Tokyo, Japan). Three digital images were captured for each blastocyst using Chronos software with a CCD camera (CS230B, Olympus, Tokyo, Japan). One image focused on the equatorial plane of the blastocyst, while the other two focused on the TE cells at the top and bottom of the blastocyst. Blastocyst measurements were taken using a straight line or a polygon tool using a software (Image J software, Version 1.6, USA). The blastocyst diameter was determined as the average of two perpendicular diameters in the equatorial plane. The blastocyst area was measured by outlining the entire blastocyst with the polygon tool in the software, as described by others [16,17,18,19]. These measurements were taken from the outer borders of the TE, excluding the area occupied by the zona pellucida (Figure 1 and Figure 2). Measurements were recorded for each transferred blastocyst, and the data were analyzed in relation to the CPR. All the measurements were performed by the same embryologist (RS). None of the parameters analyzed, including age, number of oocytes collected, BMI, endometrial thickness, cause and duration of infertility, showed any differences between the groups categorized by blastocyst diameter (≥170 µm versus <170 µm) or blastocyst area (≥25,000 µm^2^ versus <25,000 µm^2^).

### 2.4. Statistical Analysis

Data were described as numbers and percentages, where appropriate. Statistical analysis was performed with a chi-square test to compare percentages using the Statistical Package for Social Science, version 19.0. Differences were considered statistically significant at a *p*-value threshold of <0.05.

## 3. Results

In the current study, we analyzed 665 ICSI cycles that underwent eSBT on day 5. The overall median age of female patients was 33.6, and the mean number of COCs retrieved was 11.6. Out of these 665 single embryo transfers, 430 (64.7%) involved a blastocyst with a diameter ≥ 170 µm, while 235 (35.4%) with a diameter < 170 µm. When analyzing the blastocyst area, 325 (48.9%) involved a blastocyst with an area ≥ 25,000 µm^2^, while 340 (51.1%) had an area < 25,000 µm^2^. Patient characteristics are illustrated in Table 1 and Table 2. None of the parameters analyzed, including age (33.5 years for the group with diameter ≥ 170 µm versus 33.7 years for the group with a diameter < 170 µm), number of oocytes collected (11.1 oocytes: diameter ≥ 170 µm versus 12.1 oocytes: diameter < 170 µm), BMI, or cause and duration of infertility, differed between the two groups (diameter ≥ 170 µm versus <170 µm). Regarding the blastocyst area, age was similar between the two groups (33.5 years for the ≥25,000 µm^2^ group versus 33.6 years for the <25,000 µm^2^ group). The number of oocytes collected was also comparable: a mean of 12.2 oocytes for the larger area group (≥25,000 µm^2^) versus 11.0 oocytes for the smaller blastocyst area group (<25,000 µm^2^). Additionally, BMI, endometrial thickness, and cause of infertility showed no significant differences between the groups categorized by blastocyst area (≥25,000 µm^2^ versus <25,000 µm^2^). The blastocyst measurements were performed quickly on the morning of day 5, before the embryo transfer. Once the blastocyst image was captured at 400× magnification using an inverted microscope (IX71, Olympus, Tokyo, Japan), the area and diameter were calculated using the polygon tool in the software [16,17,18], and the system provided the value instantaneously. These values were then recorded either on paper or in an Excel sheet and compared to the pregnancy results once available. The results (Table 3) show that women who received a transfer of a blastocyst with a diameter ≥ 170 µm had significantly higher IR and CPR compared to those who received a blastocyst with a diameter < 170 µm (IR: 68.8% versus 36.6%, *p* < 0.001; CPR: 66.3% versus 35.7%, *p* < 0.001). When analyzing the blastocyst area, we found that 325 (48.9%) blastocysts had an area ≥ 25,000 µm^2^, while the remaining 340 (51.1%) had an area < 25,000 µm^2^. An increase in blastocyst area was positively associated with higher IR and CPR (Table 4). Specifically, when the eSBT involved a blastocyst with an area ≥ 25,000 µm^2^, it was correlated with a statistically significant higher IR and CPR compared to transfers of blastocysts with an area < 25,000 µm^2^ (IR: 69.8% versus 47.9%, *p* < 0.001; CPR: 65.5% versus 45.9% *p* < 0.001). Similar spontaneous abortion rates were observed between the two groups. According to the International Glossary on Infertility and Fertility Care, 2017 [19], spontaneous abortion rate was considered when a positive pregnancy test did not result in a fetal heartbeat being visualized via ultrasound examination at about 7 weeks of gestation.

## 4. Discussion

This study found that the transfer of blastocysts with an area ≥ 25,000 µm^2^ or a diameter ≥ 170 µm was associated with a statistically significant (*p* < 0.01) increase in IR and CPR compared to the transfer of blastocysts with an area < 25,000 µm^2^ or diameter < 170 µm. Advances in embryo culture and selection techniques have enabled embryologists to increase the percentage of single embryo transfers, aiming to reduce the risks associated with multiple gestations. A morphological assessment continues to be the primary method employed by many IVF centres globally; however, this approach is subject to significant variability between operators. As a result, incorporating more objective and quantifiable parameters to help embryologists select the embryo with the highest implantation potential for transfer would represent a valuable improvement. In this context, TLM data have introduced new variables, such as morphometric and morphokinetic features, which may serve as additional markers for predicting a successful pregnancy [20,21,22,23]. Time-lapse technology allows the detection of features that cannot be observed with standard incubation, such as reverse or abnormal cleavage [32,33,34], as well as events at the morula stage [35,36,37,38], and blastocoel collapse at the time of blastocyst formation [39,40,41,42,43]. Several studies have shown that spontaneous collapse events can be considered negative markers of embryo quality, impairing implantation and pregnancy outcomes [39,40,41,42]. The primary objective of the current study was to determine whether measuring blastocyst diameter and area could predict pregnancy success following eSET. While previous studies have assessed blastocyst expansion using grading systems rather than a morphometric measurement [44,45,46,47,48], Thompson and co-authors [49] revealed that after fresh embryo transfer, the extent of blastocyst expansion was an independent predictor of live birth outcomes. The authors reported that the grade of expansion is correlated to the blastocyst area, which aligns with our results, showing that increased blastocyst area (≥25,000 µm^2^) is associated with a statistically significant higher pregnancy rate compared to transfer of blastocysts with an area < 25,000 µm^2^). These findings are consistent with Shebi and collaborators [50], who revealed that embryos with a delayed development, which did not reach the blastocyst stage by day 5 were less likely to result in a successful pregnancy and live birth. Conversely, embryos that developed rapidly and reached the blastocyst stage by day 4 were more likely to yield higher pregnancy outcomes [20]. The morphometric blastocyst assessment has also been explored in frozen embryo transfer (FETs) by other authors [17,25,43,51,52,53]. Utsuno and collaborators, in a cross-sectional study involving 585 vitrified blastocysts, focused on the ICM area. Their findings suggested that blastocysts with an ICM area > 4500 µm^2^ had an increased IR of 45%, while those with an ICM area < 3800 µm^2^ had a much lower IR of 18% [43]. A further study on FETs, conducted by Zhao and co-workers, announced that the degree of the blastocoel expansion was significantly correlated with clinical pregnancy outcomes and had the ability to predict pregnancy in patients undergoing FETs [25]. These results have also been confirmed by another group [17]. An additional aspect currently being explored by several researchers is the chromosomal status of embryos [54,55,56,57,58,59,60], which may play a critical role in early embryonic development. Determining whether there is a connection between an embryo’s euploid composition and its blastocyst area could be very beneficial. Irani and collaborators investigated whether blastocyst grading and expansion could predict the pregnancy outcomes in the FETs of euploid blastocysts, revealing that blastocyst expansion, morphology, and ICM grade are useful predictors of pregnancy per euploid embryo [59]. Morphological grading should thus be used to assist in the selection of euploid blastocysts. Similar results were reported in a subsequent study analyzing about 700 single FETs of euploid blastocysts. This study found that the speed of blastocyst development, as well as the evaluation of blastocyst expansion, are critically important in selecting the embryo with the highest implantation potential [60]. The chromosomal status of embryos has also been explored by Huang and colleagues [54], who demonstrated that the rates of euploidy and aneuploidy were more pronounced in regions with higher and lower blastocyst expansions, respectively. The authors found that the number of euploid blastocysts was significantly higher in blastocysts with an area > 20,000 µm^2^ compared to those with less expansion (<15,000 µm^2^). This aligns with our finding, where we observed that in pregnant women, blastocyst areas were notably larger than in those who were not pregnant. Additionally, this may explain the slight difference in the abortion rate between the two groups investigated, with the rate found to be lower, at 11.1%, in the group with a larger blastocyst area (≥25,000 µm^2^), compared to 14.4% in the group with a smaller area (<25,000 µm^2^). However, this difference did not reach statistical significance, which may be due to the relatively small sample size of the cycles investigated. It is also important to note that the spontaneous abortion rate is multifactorial and may be influenced by several other factors not investigated in the current study. It seems evident that blastocyst expansion and area can be considered critical indicators of embryo development, viability, and implantation potential. Ahlstrom and co-workers [61] found that blastocyst expansion is linked to the quality and functionality of the TE layer, which plays a vital role in osmotic regulation by preventing the loss of fluid and sodium ions from the blastocoel, while promoting the influx of ions into the cavity. This results in intracellular water accumulation, driving further expansion and ultimately leading to the herniation of the zona pellucida and blastocyst hatching. Chimote and colleagues, in a prospective study involving 146 patients undergoing their first IVF cycles, aimed to analyze the developmental potential of a spontaneously hatching blastocyst. The authors found that pregnancy outcomes are higher when a hatching blastocyst is transferred, compared to a non-hatching one [13]. Since hatching is vital for implantation, it is unsurprising that blastocyst expansion and area are directly correlated with embryo viability and clinical pregnancy outcomes [13,62,63,64,65,66]. In the current study, we also examined the measurement of blastocyst diameter, and we observed a significant correlation with IR and CPR. This is in agreement with a study published by Kato and collaborators, who found a positive correlation between blastocyst diameter and clinical pregnancy in FETs cycles [52]. In line with our findings, numerous studies have confirmed that blastocyst diameter is a key factor in predicting successful pregnancy following eSBT [19,21,22] and is significantly correlated with both TE cell count and ICM area [43]. In addition, the authors found a positive association between the blastocyst diameter and ongoing pregnancy in FETs. The debate regarding which blastocyst feature, between ICM, TE, and expansion, is most relevant in predicting pregnancy is still very active in the literature. Some authors have emphasized the importance of ICM morphology in predicting pregnancy [19,20,21,22], while others argue that TE grading is more indicative of successful pregnancy outcomes [18,52,54]. Goto and colleagues [67] investigated the degree of expansion and morphology in FETs and revealed that blastocyst expansion, rather than ICM or TE morphology, was significantly associated with clinical outcomes. A study by Du and co-workers also stated that blastocyst expansion is the most critical factor in predicting live birth following both fresh and vitrified–warmed eSBT cycles [17]. Due to the inherent subjectivity in morphological assessments of blastocyst, determining the relative significance of the three morphological parameters, ICM, TE and blastocyst expansion, in predicting pregnancy outcomes is complex [19,21]. Therefore, the simple, quantitative, and measurable blastocyst morphometric evaluation proposed in this study could help reduce inter-observer variability, providing a more objective assessment of embryo viability. In addition, the present study could serve as a training set for further validation of morphometric blastocyst assessment and could be useful for future research exploring the relationship between morphometric evaluation and chromosomal aneuploidies. However, our study has some limitations. The first to be mentioned is that it is a retrospective investigation, and the sample size of 665 cycles represents a relatively small study. Thus, our results should be validated and confirmed through a prospective or larger multicenter, randomized, controlled study. Additionally, performing the morphometric assessment requires specific equipment, such as imaging tools and software. Furthermore, no biopsied embryos were included in the study; therefore, our findings may not be applicable to embryos that undergo biopsy. Moreover, all embryos in the study were produced by ICSI, so our results may not be directly applicable to IVF embryos. Our data are only relevant for day 5 blastocysts and cannot be generalized to days 6 or 7 blastocysts. While we acknowledge that live birth rates with pediatric follow-up would be the ideal endpoint for clinical research [68], conducting a powered study with live birth as the outcome would require a substantial number of participants. In some centres, it can be challenging to track patient outcomes, especially when couples travel internationally for ART treatment. Although we recognize the value of studies that measure live birth rates, we believe that smaller studies reporting clinical pregnancy outcomes can still provide valuable insights into relevant parameters, representing an important step in clinical research. Finally, we acknowledge that there may be confounding factors not explored in this study that could affect the validity of our findings.

## 5. Possible Application of the Research and Novel Technologies

Our results in morphometric evaluations, such as blastocyst size and diameter, can enhance existing grading systems for blastocyst evaluation, support AI algorithms, and improve the identification of the most promising blastocyst for transfer, thereby boosting the likelihood of successful implantation. This is especially advantageous when several blastocysts are available for transfer, as it helps pinpoint the one with the highest potential for successful implantation. The rapid advancements in IVF have seen the application of new technologies playing an increasingly important role in improving pregnancy success rate. Microfluidics, time-lapse imaging, deep learning and artificial intelligence (AI) not only support better outcomes but also enable embryologists to standardize procedures and gather more information during the selection of the single embryo to transfer. Time-lapse technologies, for instance, allow for a continuous, non-invasive embryo assessment, removing the need to disturb embryos by removing them from the culture. This innovation allows for a detailed observation of embryonic developmental events with an unprecedented level of precision. AI, which is evolving rapidly, is now being applied across various facets of IVF, including sperm and embryo selection, as well as the evaluation of IVF personnel’s performance [69,70,71,72,73]. Physicians use AI to optimize and personalize stimulation protocols, a process expected to expand and improve implantation rates in the future. In addition, automated systems for embryo culture and selection may soon rely on non-invasive measures of embryo competency, such as morphological, morphokinetic, morphometric, or molecular analyses of culture media [74,75,76,77]. These emerging technologies, combined with the ability to standardize procedures and minimize human error, promise to bring significant benefits to ART. Furthermore, the integration of advanced microscopy technologies, such as hyperspectral and fluorescence lifetime imaging, will enhance the ability to assess not only the metabolic state of embryos, but also their epigenetic status [74,75]. As these technologies continue to evolve, they are poised to revolutionize ART by offering more precise, personalized approaches to embryo evaluation, ultimately leading to improved overall success rates and healthier pregnancies.

### Time-Lapse Technology, AI and Automatic Embryo Selection in ART

In clinical embryology, AI can assist with the automatic annotation and grading of embryos more objectively, offering the potential to standardize and optimize the embryo selection process. Currently, embryologists manually annotate features during TLM incubation, a task that is prone to variability. The historical scoring system for blastocyst assessment, originally developed by Gardner and Schoolcraft [31], considers three main features: the TE, ICM, and grade of expansion. However, despite being in use for over 20 years, the relative impact of each of these parameters on pregnancy outcomes remains a topic of debate. Additionally, this grading system is subject to significant variability between operators. A study by Bormann and co-authors investigated the key performance indicators (KPIs) of ten embryologists, revealing significant differences in grading. The authors demonstrate that AI systems can detect statistical KPIs, monitor an individual embryologist’s performance and culture conditions, and enable an early detection of adverse outcomes, such as shifts in pregnancy rates [77]. A challenge in automating embryo assessment is the reliance on high-quality training data, as AI models are trained on images manually graded by embryologists. Thereby, integrating TLM and AI-based automatic annotation could standardize the embryo selection process with a high accuracy. Several authors have recently investigated automatic, non-human-mediated embryo annotation systems [76,77,78,79,80,81,82,83,84,85]. These systems must meet criteria such as speed, accuracy, reproducibility, and the ability to identify both normal and abnormal developmental features. They need to detect morphological characteristics like uneven size, vacuoles, and granularity, as well as nuclear abnormalities like multinuclear blastomeres. Ideally, these systems should appropriately weight each feature for accurate predictions. Early studies on AI have been conducted using animal models, with the goal to enabling autonomous operations without human intervention. AI attempts at deep learning and machine learning, aimed at automating grading, involved differentiating the ICM from the TE using image segmentation with support vector machines on 2D images. AI applications have been proposed by Matos and colleagues [86], who obtained a 95% accuracy in classifying mouse blastocysts. The same research group later applied AI to assess bovine embryos [87], paving the way for AI’s use in human embryo assessment [88]. Convolutional neural networks have been developed to grade blastocyst features like ICM and TE morphology. One study using TLM data achieved 97.8% accuracy for ICM and 98.1% for TE [89]. Another approach using static images predicted blastocyst expansion, ICM, and TE with 96%, 91%, and 84% accuracy, respectively [90]. AI can also aid embryologists in selecting the single best embryo for transfer. Various statistical models and algorithms have been developed to predict implantation success by analyzing embryo morphokinetic parameters through TLM [77,79,81,83,84,85,91,92,93,94]. These parameters are closely linked to embryo quality and provide valuable insights into the potential for implantation. AI systems that utilize TLM data have been designed to predict the implantation success at different stages, such as the cleavage and blastocyst stages. For instance, a deep learning model developed by Tran and colleagues [82] aimed to predict the fetal heart rate by assessing TLM videos, proposing an automated system for embryo evaluation. In a study by Chavez-Badiola and co-workers [84], static images were used to predict implantation rates with an accuracy of 70%, but the model’s predictive value was unclear compared to traditional grading methods. A multicenter study by VerMilyea and co-authors [85] tested an AI platform using single blastocyst images and found it had an accuracy of 67.7%, with sensitivity of 71.1% and specificity of 65.3%. The platform showed a 30.8% improvement over predictions made by embryologists across 11 IVF centres. A recent study by Illingworth and colleagues aimed to investigate the value of deep learning in selecting the single embryo for transfer in ART cycles. This multicenter, randomized, double-blind study was conducted across 14 IVF clinics in Europe and Australia [95]. The trial included 1,066 patients and found that the group in which embryos were selected for transfer using AI achieved a clinical pregnancy rate of 46.5% (248 of 533 patients), compared to 48.2% (257 of 533 patients) in the morphology arm. Through this study, the authors demonstrated the validity of deep learning in predicting clinical pregnancy rates when compared to standard morphology. However, despite AI’s potential, a key question remains whether a universal system for embryo evaluation will be effective across different IVF laboratories, considering the influence of local factors such as culture and media, patient characteristics, and incubators. Additionally, incorporating clinical features like patient age, ovarian reserve, and stimulation protocols is necessary to improve predictive accuracy. Ultimately, while AI shows great promise in embryo selection, further research is needed to address the challenges related to data quality, model generalizability, and the integration of clinical factors. Finally, for laboratories utilizing automated or semi-automated embryo evaluation [96], integrating morphometric measurements, such as blastocyst area and diameter, could enhance existing grading systems and optimize the selection of the most viable blastocyst for transfer, thereby boosting the likelihood of a successful implantation and a healthy pregnancy in ART cycles.

## 6. Conclusions

This current study presents an innovative, measurable, objective, and non-invasive approach to selecting the most viable blastocyst for transfer with the highest implantation potential. The method utilizes a morphometric blastocyst assessment, which is objective, cost-effective, and easy to implement in routine embryology practices. It involves capturing digital images of the blastocyst and measuring its area and diameter—a simple and quick process. This approach can be seamlessly integrated into the existing blastocyst grading systems, providing valuable insights into the potential success of pregnancy outcomes. It is particularly beneficial when multiple blastocysts are available for transfer, as it helps identify the blastocyst with the greatest chance of successful implantation. Incorporating morphometric assessments, such as blastocyst area and diameter, could refine current grading systems, support AI algorithms, and improve the selection of the most viable blastocyst for transfer, thereby increasing the chances of a successful implantation. Overall, this approach represents a promising advancement in optimizing blastocyst selection for ART and improving the chances of achieving a healthy pregnancy.

## Figures and Tables

**Table 1:** Characteristics of patients undergoing eSET and diameters.

	Group (Diameter ≥ 170 µm)	Group (Diameter < 170 µm)	*p*-Value
Maternal age (years)	33.5 ± 3.48	33.7 ± 4.09	NS
Oocyte collected (n)	11.1 ± 1.48	12.1 ± 1.88	NS
Transferred blastocysts (n)	1	1	NS
Basal FSH (mIU/mL)	7.15 ± 1.51	7.64 ± 1.79	NS
Maternal BMI	28.67 ± 2.16	29.17 ± 2.14	NS
Infertility duration (years)	3.17 ± 1.47	3.67 ± 1.75	NS
Endometrial thickness (mm)	8.83 ± 1.47	9.10 ± 1.10	NS

Differences were considered statistically significant at the level of *p* value < 0.05. NS: not significant value.

**Table 2:** Characteristics of patients undergoing eSET and blastocyst areas.

	Blastocyst (Area ≥ 25,000 µm^2^)	Blastocyst (Area < 25,000 µm^2^)	*p*-Value
Maternal age (years)	33.5 ± 3.48	33.6 ± 4.09	NS
Oocyte collected (n)	12.2 ± 1.34	11.0 ± 1.56	NS
Transferred blastocysts (n)	1	1	NS
Basal FSH (mIU/mL)	8.66 ± 1.51	8.04 ± 1.79	NS
Maternal BMI	29.67 ± 2.16	28.45 ± 2.14	NS
Infertility duration (years)	3.87 ± 1.47	3.24 ± 1.75	NS
Endometrial thickness (mm)	9.20 ± 1.47	8.94 ± 1.10	NS

Differences were considered statistically significant at the level of *p* value < 0.05. NS: not significant value.

**Table 3:** The relation between the maximum blastocyst diameter and pregnancy outcomes.

	Blastocyst Diameter ≥170 µm	Blastocyst Diameter <170 µm	*p*-Value
Beta hCG Positive	78.8% (339/430)	48.0% (113/235)	<0.001
Implantation Rate	68.8% (296/430)	36.6% (86/235)	<0.001
Clinical Pregnancy Rate (with fetal heartbeat)	66.3% (285/430)	35.7% (84/235)	<0.001
Spontaneous abortion	12.6% (54/430)	12.3% (29/235)	NS

Differences were considered statistically significant at the level of *p* value < 0.05. NS: not significant value.

**Table 4:** The relation between the blastocyst area and pregnancy outcomes.

	Blastocyst Area ≥25,000 µm^2^	Blastocyst Area <25,000 µm^2^	*p*-Value
Beta hCG Positive	76.6% (249/325)	60.3% (205/340)	<0.001
Implantation Rate	69.8% (227/325)	47.9% (163/340)	<0.001
Clinical Pregnancy Rate (with fetal heartbeat)	65.5% (213/325)	45.9% (156/340)	<0.001
Spontaneous abortion	11.1% (36/325)	14.4% (49/340)	NS

Differences were considered statistically significant at the level of *p* value < 0.05. NS: not significant value.

## Data Availability

The original contributions presented in this study are included in the article. Further inquiries can be directed to the corresponding author.
